# Effect of Age on Blood Glucose and Plasma Insulin, Glucagon, Ghrelin, CCK, GIP, and GLP-1 Responses to Whey Protein Ingestion

**DOI:** 10.3390/nu10010002

**Published:** 2017-12-21

**Authors:** Caroline Giezenaar, Amy T. Hutchison, Natalie D. Luscombe-Marsh, Ian Chapman, Michael Horowitz, Stijn Soenen

**Affiliations:** 1 Discipline of Medicine and National Health and Medical Research Council of Australia (NHMRC) Centre of Research Excellence in Translating Nutritional Science to Good Health, Adelaide Medical School, 5000 Adelaide, Australia; caroline.giezenaar@adelaide.edu.au (C.G.); amy.hutchsison@adelaide.edu.au (A.T.H.); natalie.luscombe-marsh@csiro.au (N.D.L.-M.); ian.chapman@adelaide.edu.au (I.C.); michael.horowitz@adelaide.edu.au (M.H.); 2 Commonwealth Scientific and Industrial Research Organisation (CSIRO), Food and Nutrition, 5000 Adelaide, Australia

**Keywords:** age-effect, whey protein, gut hormones

## Abstract

Protein-rich supplements are used widely to prevent and manage undernutrition in older people. We have previously shown that healthy older, compared to younger, adults have less suppression of energy intake by whey protein—although the effects of age on appetite-related gut hormones are largely unknown. The aim of this study was to determine and compare the acute effects of whey protein loads on blood glucose and plasma gut hormone concentrations in older and younger adults. Sixteen healthy older (eight men, eight women; mean ± SEM: age: 72 ± 1 years; body mass index: 25 ± 1 kg/m^2^) and 16 younger (eight men, eight women; 24 ± 1 years; 23 ± 0.4 kg/m^2^) adults were studied on three occasions in which they ingested 30 g (120 kcal) or 70 g (280 kcal) whey protein, or a flavored-water control drink (~2 kcal). At regular intervals over 180 min, blood glucose and plasma insulin, glucagon, ghrelin, cholecystokinin (CCK), gastric inhibitory peptide (GIP), and glucagon-like peptide-1 (GLP-1) concentrations were measured. Plasma ghrelin was dose-dependently suppressed and insulin, glucagon, CCK, GIP, and GLP-1 concentrations were dose-dependently increased by the whey protein ingestion, while blood glucose concentrations were comparable during all study days. The stimulation of plasma CCK and GIP concentrations was greater in older than younger adults. In conclusion, orally ingested whey protein resulted in load-dependent gut hormone responses, which were greater for plasma CCK and GIP in older compared to younger adults.

## 1. Introduction

Despite the well-recognized major adverse impact of nutritional impairment on the health of the elderly, including ageing-related muscle loss [1], and related increase in the use of high-energy drinks, usually rich in whey protein, few nutritional studies have involved older people. We have recently reported that healthy older, compared to younger, adults have less suppression of energy intake by whey protein, ether ingested orally [2] or infused directly into the proximal small intestine [3].

Appetite, energy intake, and blood glucose regulation are likely to be dependent on gastrointestinal mechanisms triggered by the interaction with the nutrients ingested. Mechanisms which reduce energy intake in younger adults include the stimulation of gut hormone secretion, e.g., cholecystokinin (CCK) and glucagon-like peptide (GLP-1), and the suppression of ghrelin. The incretin hormones gastric inhibitory polypeptide (GIP) and GLP-1 play major roles in the control of plasma insulin, glucagon, and blood glucose concentrations in response to nutrient ingestion [4]. We, and others, have reported that age affects gut hormone responses; healthy older, compared to younger, adults had higher CCK concentrations after overnight fasting, after mixed nutrient intake [5,6], and during intraduodenal glucose and lipid infusions [7], in addition to higher insulin in response to intraduodenal glucose infusion [8], higher GIP after glucose ingestion [9,10], and higher GLP-1 after an overnight fast [9,11,12] as well as after glucose [9] and mixed macronutrient intakes [11], while the reported effects of age on fasting and postprandial ghrelin after mixed macronutrient intakes are inconsistent [6,12,13,14,15].

The aims of the study were to further determine the effects of oral whey protein loads on blood glucose and plasma insulin, glucagon, ghrelin, CCK, GIP, and GLP-1 concentrations in older as well as younger adults. We hypothesized that orally administered whey protein would result in load-related responses of glucose and gut hormones, and that these responses to whey protein would be greater in older than younger subjects.

## 2. Materials and Methods

### 2.1. Subjects

Sixteen older adults (eight men and eight women, age: mean ± standard error of the mean (SEM): 72 ± 1 years; body weight: 70 ± 3 kg; height: 1.66 ± 0.02 m; body mass index (BMI): 25 ± 1 kg/m^2^) and 16 younger adults (eight men and eight women, 24 ± 1 years; 68 ± 2 kg; 1.71 ± 0.02 m; 23 ± 0.4 kg/m^2^) were included. The study protocol was approved by the Royal Adelaide Hospital Research Ethics Committee, and subjects provided written informed consent (clinical trial registration: ACTRN12612000941864, www.anzctr.org.au).

### 2.2. Protocol

The protocol was identical to that of our previous studies comparing younger and older men [2], and older men and women [16]—results of blood glucose and plasma gut hormone concentrations in the healthy older women compared to men are published [16]. The study had a randomized (using the method of randomly permuted blocks; www.randomization.com (16 subjects randomized in one block with random permutations)) double-blind cross-over design including three study days, separated by three to 14 days.

Subjects consumed a standardized evening meal (beef lasagna (McCain Foods Pty Ltd., Wendouree, VIC, Australia), ~591 kcal) before the study days at ~19.00 h. They were instructed to fast overnight from solids and liquids thereafter and to refrain from strenuous physical activity. On the study day, subjects attended the laboratory at ~08.30 h and were seated in an upright position [2,16].

Subjects ingested drinks containing 30 g (120 kcal) or 70 g (280 kcal) whey protein or a control drink (~2 kcal) [2,16]. The drinks were prepared by a research assistant who was not involved in the data analysis of the study results, flavored with diet lime cordial (Bickford’s Australia Pty Ltd., Salisbury South, SA, Australia), and served in a covered cup.

### 2.3. Measurements

Blood samples were collected, using an intravenous cannula, at 0, 5, 15, 30, 45, 60, 90, 120, 150, and 180 min, into ice-chilled ethylenediaminetetraacetic acid (EDTA) coated tubes. No inhibitors were added [17]. Plasma was obtained by centrifugation for 15 min at 3200 rpm at 4 °C and samples were stored at −80 °C. Ad libitum energy intake (kcal) was determined from a buffet-style meal (180–210 min) [2]. Gastric emptying was determined from total gastric volume measurements by three-dimensional (3D) ultrasonography (Logiq™ 9 ultrasound system, GE Healthcare Technologies, Sydney, NSW, Australia) [2].

Blood glucose (millimoles per liter) was determined immediately after collection by the glucose oxidase method using a portable glucometer (Optium Xceed, Abbott Laboratories, Doncaster, VIC Australia). Intra- and inter-assay coefficients of variation were 3.2 and 10.8%. Plasma total insulin (milliunits per liter (mU/L)) was measured by enzyme-linked immunosorbent assay (ELISA) immunoassay (10-1113; Mercodia, Uppsala, Sweden). The minimum detectable limit was 1.0 mU/L. Intra- and inter-assay coefficients of variation were 3.0% and 8.7%. Plasma total glucagon (picogram per milliliter (pg/mL)), ghrelin (pg/mL), CCK-8 (picomoles per liter (pmol/L)), GIP (pmol/L), and GLP-1 (pmol/L) were measured by radioimmunoassay (RIA) [16]. Minimum detectable limits were 20 pg/mL, 40 pg/mL, 1 pmol/L, 2 pmol/L, and 3 pmol/L. Intra- and inter-assay coefficients of variance were: insulin: 3.0% and 8.7%, glucagon: 4.3% and 7.1%, ghrelin: 6.7% and 12.1%, CCK: 5.4% and 13.9%, GIP: 3.9% and 9%, GLP-1: 6.3% and 10.3%.

### 2.4. Data and Statistical Analysis

Sixteen subjects per age group would allow detection of differences in the area under the curve (AUC) of the primary outcomes of 25,920 pg/mL min ghrelin, 198 pmol/L min CCK, and 1080 pmol/L min GLP-1 between groups with power equal to 0.8 and alpha to 0.05. Statistical analyses were performed using SPSS software (version 22; IBM, Armonk, NY, USA). Effects of age and protein load and their interaction effect were determined using a repeated measures mixed-effect model, including baseline values as a covariate and Bonferroni’s post hoc correction. AUC was calculated from baseline to 180 min using the trapezoidal rule and peak/nadir as the largest change from baseline. Statistical significance was accepted at *p* < 0.05. All data are presented as means ± SEMs.

## 3. Results

Baseline concentrations after an overnight fast of blood glucose (mean ± SEM; older and younger: 5.4 ± 0.1 and 5.4 ± 0.1 mmol/L), plasma glucagon (64 ± 4 and 68 ± 4 pg/mL), ghrelin (1438 ± 156 and 1507 ± 207 pg/mL), and GIP (13 ± 2 vs. 16 ± 2 pmol/L) were comparable between age groups (*p* > 0.05), while insulin was lower (older vs. younger: 3.3 ± 0.4 vs. 5.3 ± 0.6 mU/L, *p* = 0.006) and CCK (4.8 ± 0.6 vs. 3.3 ± 0.4 pmol/L, *p* = 0.033) and GLP-1 (32 ± 4 vs. 22 ± 2 pmol/L, *p* = 0.041) were higher in healthy older compared to younger adults.

**Figure 1:**
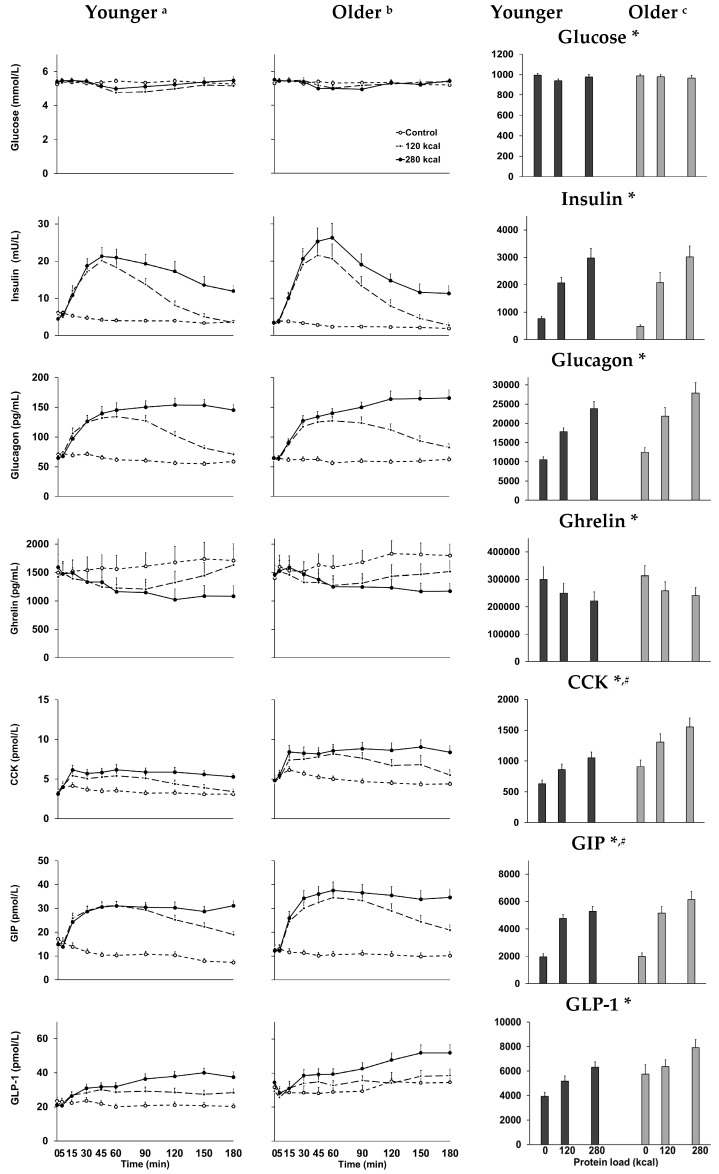
^a,b^ Mean and ^c^ area under the curve ± SEM blood glucose and plasma insulin, glucagon, ghrelin, CCK, GIP, and GLP-1 concentrations in younger (*n* = 16) and older (*n* = 16) adults after 30 g (120 kcal; dashed line with closed circles) or 70 g (280 kcal; solid line with closed circles) whey protein ingestion, or control (~2 kcal; dashed line with open circles). Effects of age and protein load as well as the interaction effect of age by protein load were determined using a mixed-effect model with baseline concentrations as covariates. * *p* < 0.05, protein load effect: AUC glucose and ghrelin dose-dependently decreased, while insulin, glucagon, CCK, GIP, and GLP-1 dose-dependently increased. # *p* < 0.05, age effect: healthy older, compared to younger, adults had higher AUC plasma, CCK, and GIP concentrations.

AUC ghrelin dose-dependently decreased and insulin, glucagon, CCK, GIP, and GLP-1 dose-dependently increased (Figure 1, post hoc effects: 30 g and 70 g vs. control, 70 g vs. 30 g protein drink, all *p* < 0.01). Nadir glucose was lower (*p* = 0.005), and peak glucagon (*p* = 0.001) and GLP-1 (*p* = 0.001) were higher after 70 g compared to 30 g whey protein ingestion. Time to peak of glucagon was higher after 70 g compared to 30 g whey protein ingestion (*p* < 0.001). Plasma gut hormone concentrations were related (ghrelin positively, and insulin, glucagon, CCK, GIP, and GLP-1 negatively) to energy intake (energy intake after 0 g, 30 g, and 70 g whey protein intake: younger: 1082 ± 106 kcal, 963 ± 79 kcal, 948 ± 82 kcal; older: 843 ± 77 kcal, 803 ± 75 kcal, 793 ± 78 kcal) and gastric emptying (younger and older men [2] and older men and women [16] published previously; Table 1).

Older compared to younger adults had higher AUC and peak concentrations and time to peak of CCK (AUC: *p* = 0.031; older vs. younger: peak: 5.0 ± 0.7 pmol/L vs. 3.3 ± 0.3 pmol/L, *p* = 0.007; time to peak: 95 ± 12 min vs. 65 ± 8 min, *p* = 0.046) and GIP (AUC: *p* = 0.036; peak: 17.8 ± 2.7 pmol/L vs. 16.6 ± 1.4 pmol/L, *p* = 0.028; time to peak: 132 ± 11 min vs. 101 ± 9 min, *p* = 0.037). AUC interaction effects of age by protein load were not significant.

## 4. Discussion

This study examined the influence of age on the acute effects of orally ingested whey protein on blood glucose and plasma gut hormone concentrations in healthy adults. Plasma ghrelin was dose-dependently suppressed, while insulin, glucagon, CCK, GIP and GLP-1 concentrations were dose-dependently increased by the whey protein ingestion. Our observations extend the previously reported data of the acute effects of orally ingested whey protein on plasma insulin, glucagon, ghrelin, CCK, GIP, and GLP-1 concentrations in young adults [18,19]. The protein load effects were particularly evident after ~60 min, when the majority of the dose of 30 g whey protein had emptied from the stomach [16]; plasma concentrations returned to baseline after 30 g, while they remained at their maximal increase/decrease after 70 g whey protein intake.

Our findings confirmed earlier reports that older, when compared to younger, adults have higher plasma CCK [5,6,7] and GLP-1 [9,11] concentrations after an overnight fast, while fasting insulin concentrations were reduced in our study in healthy adults. Age also affected CCK and GIP, but not insulin, responses following whey protein ingestion; as previously reported after mixed macronutrient ingestion for CCK [5,6] and oral, but not intraduodenally infused [20], glucose ingestion for GIP [9,10], glucose, and insulin [8,20], postprandial concentrations were greater. The higher plasma CCK and GIP concentrations in older rather than younger adults may be related to differences in the small intestinal transit of the whey protein, and clearance including GIP inactivation by dipeptidyl peptidase IV (DPP-IV) and renal processes [9]. The higher incretin hormone GIP response following whey protein ingestion in older compared to younger adults is likely to be beneficial for glycemic control in older people.

The causes of the age-related reduction in the suppression of energy intake by nutrients observed in this and other studies must include altered responses to the presence of nutrients in the small intestine, because the reduced suppression is observed after intraduodenal [3] as well as oral nutrient administration [2,6,21]. CCK is a anorexigenic hormone and acts to suppress hunger and food intake [22]. We have reported previously that older, when compared to younger, age in healthy subjects is associated with at least preserved, and possibly even increased, sensitivity to the satiating effects of exogenously administered CCK [23]. Because plasma fasting and post-protein CCK concentrations were higher in older compared to young subjects in the present and previous studies, it is perhaps surprising that these higher concentrations were associated with reduced, not increased, protein-induced suppression of energy intake in the healthy older compared to young adult subjects [2,3]. It is possible that the test meal may have been given too late at 3 hours to assess the full effect of CCK changes, as plasma concentrations had returned to baseline by then after all but the highest whey protein load. Nevertheless, these findings are consistent with our previous finding that under-nourished older people have higher fasting and post-nutrient CCK concentrations in comparison to well-nourished older people, but reduced nutrient-induced suppression of food intake compared to well-nourished older people [6]. Together, these findings suggest that age-related changes in CCK (circulating concentrations and/or action) are unlikely to contribute much, if anything, to the age-related reduction in food intake after the ingestion of protein and other nutrients.

The findings of this study do not exclude a role for GLP-1 or GIP in the lesser suppression of food intake by whey protein in healthy older subjects. Baseline circulating concentrations of the anorexigenic hormone GLP-1 were significantly higher in older compared to younger subjects, with no difference between age groups in the subsequent whey protein-induced rise, consistent with responses during intraduodenal infusions of lipid and glucose [7]. The higher baseline GLP-1 levels may have acted to further inhibit the suppression of appetite and thus food intake after whey protein ingestion. GLP-1 is mainly secreted more distally in the gastrointestinal tract (i.e., ileum and colon) than CCK and GIP (expressed mainly in the duodenum and jejunum), and the GLP-1 concentrations following whey protein ingestion increased more slowly than CCK and GIP concentrations. The emptying of food content from the stomach is slowed down by feedback mechanisms in the intestines including the release of CCK and GLP-1 [24,25]; indeed, gastric emptying of the whey protein was slower in the older compared to younger adults [2]. Although the effect of GIP on human appetite and food intake, if any, is not clear, there is limited animal evidence to suggest it may act to stimulate food intake; GIP receptor-deficient mice are resistant to diet-induced obesity [26]. The greater increase in circulating GIP concentrations after whey protein in healthy older compared to younger subjects might therefore act to reduce the protein-induced suppression of food intake. More studies will be required to investigate the role of these hormones in age-related feeding changes. Also, psychological factors, including increased dietary restraint, particularly in women [27], may affect the short-term energy intake regulation of older adults.

Healthy older and younger adults had comparable plasma ghrelin concentrations following whey protein ingestion, consistent with responses to mixed-nutrient intake in some [12,15] but not all previous studies [6,13,14]. It has been suggested that aging-related changes in body composition (i.e., a decrease in lean mass and increase in fat mass) may act to decrease fasting [28] and postprandial [6] ghrelin concentrations, as body fat is negatively correlated to ghrelin concentrations [29] and tends to increase with older age. Other studies, however, have found higher postprandial and fasting ghrelin concentrations in older adults than those in younger adults and impaired suppression of ghrelin after the consumption of a mixed-nutrient meal in older compared to younger subjects [13,14].

This study has several limitations, including the relatively small subject numbers. Total ghrelin instead of active ghrelin was measured, which could be considered to be less than optimal; however, the results appeared to be clear-cut, with significant dose-dependent suppressive effects of the protein loads on ghrelin in the direction expected.

## 5. Conclusions

The finding that plasma gut hormone responses to whey protein are not blunted in healthy older compared to younger men is likely to have implications to the composition of dietary supplements for older people, and warrants further research to their relation to food intake and glycemic control in older people.

## Figures and Tables

**Table 1:** Correlations between gut hormones, energy intake, and gastric emptying.

	Energy Intake		Gastric Emptying (T50)	
	*r*	*p*	*r*	*p*
Glucose	0.05	0.68	−0.14	0.28
Insulin	−0.41	0.001	0.80	<0.001
Glucagon	−0.34	0.005	0.81	<0.001
Ghrelin	0.36	0.003	−0.53	<0.001
CCK	−0.33	0.008	0.77	<0.001
GIP	−0.32	0.008	0.75	<0.001
GLP-1	−0.31	0.011	0.68	<0.001

*r* and *p* values of within-subject correlations between energy intake (0 g (control, ~2 kcal), 30 g (120 kcal) and 70 g (280 kcal) whey protein intake: younger *n* = 16: 1082 ± 106 kcal, 963 ± 79 kcal, 948 ± 82 kcal; older *n* = 16: 843 ± 77 kcal, 803 ± 75 kcal, 793 ± 78 kcal), gastric empting half time (T50; control, 30 and 70 g whey protein intake: younger *n* = 16: 16 ± 1 min, 32 ± 3 min, 85 ± 10 min; older *n* = 15: 23 ± 2 min, 65 ± 7 min, 130 ± 10 min) and blood glucose (mmol/L), plasma insulin (mU/L), glucagon (pg/mL), ghrelin (pg/mL), CCK (pmol/L), GIP (pmol/L), and GLP-1 (pmol/L) concentrations (AUC 0–180 min) in healthy older and younger adults. Within-subject correlations were determined by using a general linear model with fixed slope and random intercept.
